# Neonatal pulmonary sequestration (PS) with rhabdomyomas-like hyperplasia: A case report

**DOI:** 10.1097/MD.0000000000020052

**Published:** 2020-05-15

**Authors:** Xiu-mei Liu, Li-mei Yuan, Yu-rui Wu, Chun-ju Zhou

**Affiliations:** a Qilu Children's Hospital of Shandong Universities; b The Fifth People's Hospital of Jinan, Jinan, Shandong, China.; c Children's Hospital of Capital Medical University, Beijing, China.

**Keywords:** pulmonary sequestration (PS), rhabdomyomas hyperplasia, neonatal

## Abstract

**Introduction:**

Pulmonary sequestration (PS) is a rare pulmonary congenital malformation characterized by disconnection with the tracheobronchial tree or the pulmonary arterial blood supply thus impeding the connection to the arterial blood supply from systemic circulation, ultimately resulting in a non-functional lung.

**Patient concerns:**

A 73-day-old boy with rhabdomyomatoid hyperplasia was hospitalized for cough and fever 2 months after birth.

**Diagnoses:**

Routine B-ultrasound revealed a cystic malformation in the right lung. CT showed increased volume of the right lung accompanied with cystic low-density shadows of different sizes and a blood vessel leading from the abdominal aorta into the lesion lung. Thus, he was diagnosed with PS.

**Interventions:**

The boy underwent an entire lobectomy of the lower lobe of the right lung. The operation field revealed several malformed blood vessels from the apposition to the right lower lobe. Most of the lung had cystic adenomatoid malformations. Other parts consisted of well-differentiated cystic dilated bronchus and striated muscles. Histopathological examination revealed rhabdomyus fibroblasts distributed throughout the bronchi. Nuclei were located at the margins of the cells. Striated muscle fibers observed as immunological markers (myogenin and desmin) of striated muscles were positively detected.

**Outcomes:**

The boy recovered uneventfully after his operation and no abnormal clinical findings were found at the 10-month follow-up.

**Conclusion:**

PS can not only accompany other developmental abnormalities, but can also associate with rhabdomyomatoid hyperplasia. This lesion is not a fatal congenital malformation and instead is a benign lesion affecting only a single lobe. It has a relatively good prognosis.

## Abstract 2

Supplemental Digital Content is available in the text

## 1 Introduction

Pulmonary sequestration (PS) is a special type of rare pulmonary congenital malformation characterized by disconnection with the tracheobronchial tree or the pulmonary arterial blood supply thus impeding the connection to the arterial blood supply from the systemic circulation, ultimately resulting in a non-functional lung. Based on the localization, PS is classified into 2 types: intralobar and extralobar. The PS located within the normal pulmonary visceral pleura is intralobar PS, while the PS located outside of the pulmonary visceral pleura is extralobar.^[1]^ PS cases with rhabdomyomas hyperplasia are rarer with only 17 cases reported to date.^[2]^

## 2 Case report

A 73-day-old boy, a second child, was born after a normal, full-term pregnancy and delivery. His birth weight was 3.65 kg; there were no records of suffocation after birth. His parents were healthy with no overlap in family trees and no reports of any familial inherited disorders. Routine B-ultrasound examination at 5 months gestation revealed a cystic malformation in the right lung. Two months and 7 days after birth, he started to cough, and suffered from nocturnal cough, asthma, and fever (<38.9°C) without any other obvious causes.

**Figure 1:**
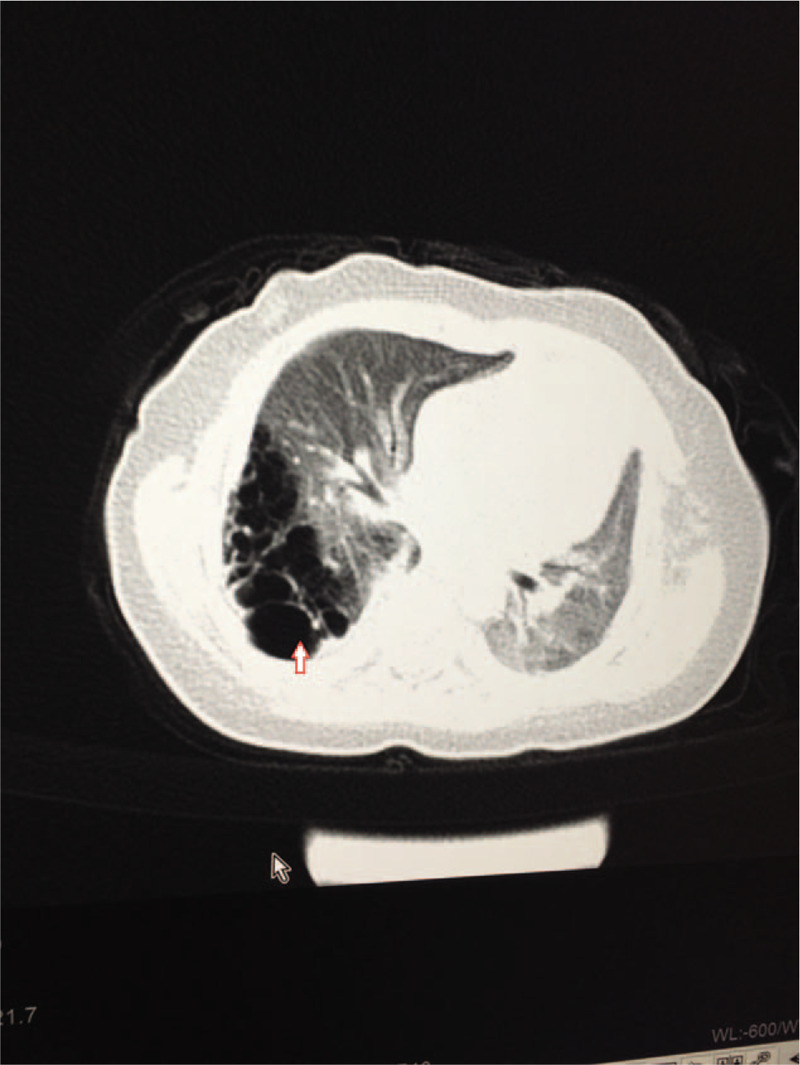
Chest CT examination at cross section of eighth thoracic vertebra-position after hospitalization. CT examination showed an increased volume in the right lung (especially at the lower lobe) with a large soft tissue density shadow at the lower leaf, non-uniform internal density without any bronchial air sign, and a cystic low-density shadow (arrowed) with clustered distribution of thick uneven wall. Mediastinum leftward shift to compress the left lung.

At the check-in examination, his body temperature, pulse, respiratory frequency, and weight were 36.8°C, 118 beats/min, 32 beats/min, and 5.7 kg, respectively. His breathing was stable and regular during examination. Trachea located at center. White blood cell counts, red blood cell counts, hemoglobin level, and platelet counts were 6.20 × 10^9^/L, 3.28 × 10^12^/L, 93 g/L, and 230 × 10^9^/L, respectively. Hepatic and renal function tests were normal. He was diagnosed as congenital PS in the lower lobe of the right lung with congenital cystic adenomatoid malformations and pneumonia in the right lower lobe based on a chest CT [Supplemental Figure 1 (Supp Fig. 1)]. The infant then underwent an entire lobectomy of the lower lobe of the right lung. Histopathological analysis of the excised lung tissues (Supp Fig. 2–5) revealed a cystic adenomatoid-like malformation. The other part of the solid area consisted of dilated bronchus and well-differentiated striated muscle fibers. The striated muscle fibers were clearly visible and stacked parallel or bundled around the dilated bronchus. Well-differentiated striated muscle fibers in horizontal stripes showed a prominent transverse streak with the nucleus located on the surface of each striated muscle cell. However, the nucleus of the striated myofibroblasts was in the cytoplasm of the myofibroblasts. Immunohistochemical staining clearly revealed striated myogenin and sarcomeric actin without any smooth muscle actin. The infant had an uneventful recovery and was postoperatively healthy without any apparent abnormal clinical evidence at the 10-month follow-up (Fig. 1).

## 3 Discussion and conclusions

PS is often accompanied by pulmonary airway developmental malformations, such as pulmonary hypoplasia, pleurisy pleural effusions, diaphragmatic hernia, cardiac dysplasia, and intestinal development malformations. Diesen and Megison^[1]^ reported a case of PS (with herniation sac) who had dyspnea at birth. Yusef et al^[3]^ reported 1 case of PS with pulmonary echinococcosis. Miyagi et al^[4]^ reported a female PS infant with an extralobar type PS accompanying esophageal hiatal hernia in a posterior mediastinal mass discovered by CT scan 2 weeks before birth. Kuo et al^[5]^ showed an intralobar type PS with abdominal bulging. Herbert et al ^[6]^ presented a PS patient with complex PS who was successfully treated using arterial embolization. Among previous reports, this is only complex PS case that had a successful surgical resection. PS cases with rhabdomyomatoid hyperplasia are rarer. The case reported here is a neonate PS with rhabdomyomatoid hyperplasia. CT scan showed an abnormal blood vessel originating from the abdominal aorta that supplied the right lung lobe. Histopathology examination of the lung tissue clearly revealed the typical characteristics of striated muscle cells rather than cardiomyocytes and smooth muscle (Supp Fig. 3–5).

Chen et al reported 1 case of neonatal pulmonary rhabdomyomas hyperplasia with dyspnea, severe left ventricular dysplasia, cleft palate, facial deviation, jaw contraction, bilateral hip dislocation, and other systemic developmental malformations. Hardisson et al^[2]^ summarized 16 cases of infantile rhabdomyomas hyperplasia, including 6 cases of stillbirth autopsy, 8 cases of neonate, and 2 cases of infants; 10 of the cases had lung development malformations, 4 had pulmonary hypoplasia, and the other 2 cases were without pulmonary deformity. Microscopic examination showed large accumulation of striated muscle fibers parallel to or bundled around the dilated bronchus covered in cysts with respiratory epithelium.

Pulmonary rhabdomyomatosis properly describes the rare accumulation of striated muscle fibers in the lung interstitium. Histologically, no skeletal muscle tissue should be in the trachea and lung. The exact mechanism of skeletal muscle tissue distribution in trachea and lung in rhabdomyomatoid hyperplasia remains unknown. An in vitro embryonic developmental study^[2]^ showed that the pluripotent stem mesenchymal cells derived from the foregut have the potential to develop into striated muscle cells, and that striated muscle cell differentiation is likely to be related to the differential imbalance of the epithelial and mesenchyme lining in lung embryos. This misleading developmental malformation presented as a cystic adenomatoid malformation which manifested as different-sized cystic cavities lining the ciliated epithelium, some visible striated muscle cells, and no cartilage components.

A PS diagnosis relies on imaging to reveal abnormal blood vessels from the aorta that supply the diseased lung tissue. Three-dimensional spiral CT angiography is a non-invasive examination technique that has become the preferred method for diagnosing PS.^[7]^ A diagnosis of PS accompanied by rhabdomyomatoid hyperplasia depends on histological observation and immunohistochemical marker examination under the microscope.

PS presenting with other developmental abnormalities or rhabdomyomas hyperplasia need to be identified with cystic bronchiectasis, lung abscess, bullae, and cystic pleuropulmonary blastoma. Those disorders do not present with any abnormal systemic vascular supplements in imaging examinations. Cystic pleuropulmonary blastoma contains markedly undifferentiated immature striated myofibroblasts, immature cartilaginous islands, epithelium, and abundant mucus matrix. Once the diagnosis of pediatric PS is clear, with or without clinical symptoms, surgical treatment should be performed as soon as possible. The surgery should be performed after any infection has resolved. In principle, the lobectomy should be performed for intralobar PS, while isolating lung resection can be used for extralobar PS.

Since the blood supply of PS is from the systemic circulation, abnormal blood vessels should be gently isolated with great caution to avoid accidental injury of abnormal blood vessels that can result in fatal bleeding.^[7]^ Currently, some scholars^[6]^ advocate the application of embolization therapy for asymptomatic pediatric PS patients. However, embolization carries major risks such as infection and tumorigenesis so selection of embolization therapy for PS patients remains controversial.^[8]^

PS can not only accompany other developmental abnormalities, but can also associate with rhabdomyomatoid hyperplasia. Most importantly, this lesion is not a fatal congenital malformation, instead it is a benign lesion affecting only a single lobe and it has a relatively good prognosis. Children with recurrent respiratory tract infections accompanied by dysplasia in other sites should receive a lung imaging examination. Children diagnosed with PS can be cured by surgical removal of the diseased lung tissue, but proper monitoring and identification of those patients are crucial to the curative process.

## Author contributions

L.X. was involved in all of the study process, collected and analyzed all the clinical information, analyzed the histochemical stains, and wrote the manuscript; Y.L. reviewed, directed, and modified the manuscript; W.Y. provided some clinical information (including medical history and images) and was involved in manuscript preparation; and Z.C. directed some pathological techniques, reviewed and consulted the manuscript, and provided some references.

**Conceptualization:** Xiu-mei Liu, Yu-rui Wu.

**Data curation:** Xiu-mei Liu, Li-mei Yuan, Yu-rui Wu.

**Formal analysis:** Xiu-mei Liu, Li-mei Yuan, Chun-ju Zhou.

**Funding acquisition:** Li-mei Yuan, Chun-ju Zhou.

**Investigation:** Yu-rui Wu.

**Methodology:** Xiu-mei Liu.

**Software:** Xiu-mei Liu.

**Supervision:** Xiu-mei Liu.

## Supplementary Material

Supplemental Digital Content

## Supplementary Material

Supplemental Digital Content

## Supplementary Material

Supplemental Digital Content

## Supplementary Material

Supplemental Digital Content

## Supplementary Material

Supplemental Digital Content
