# Supplementary figures and images for: Neonatal pulmonary sequestration (PS) with rhabdomyomas-like hyperplasia: A case report
Source: Medicine (Baltimore). 2020 May 15;99(20):e20052. doi: 10.1097/MD.0000000000020052 (PMC7313550; doi:10.1097/MD.0000000000020052)

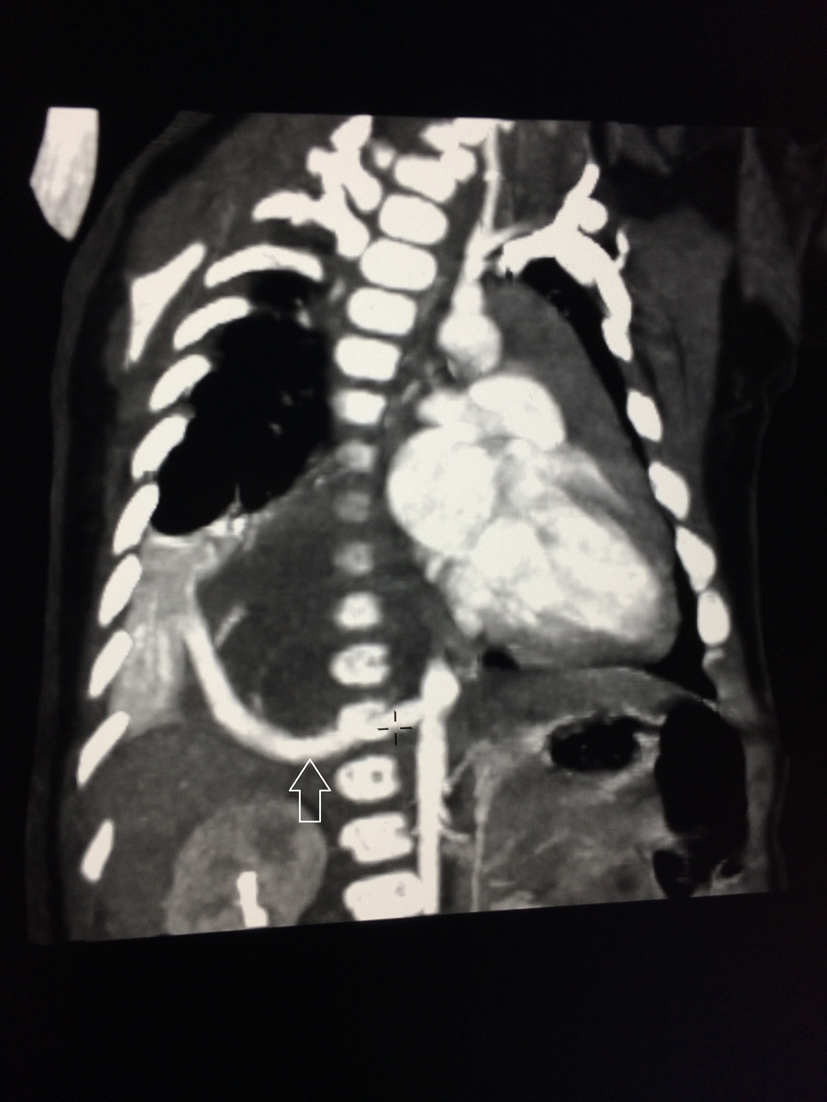

Supplement: Supplemental Digital Content [file medi-99-e20052-s001.tif]

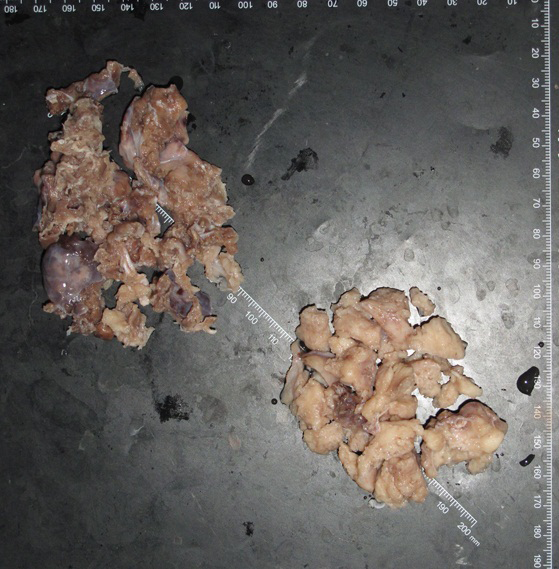

Supplement: Supplemental Digital Content [file medi-99-e20052-s002.tif]

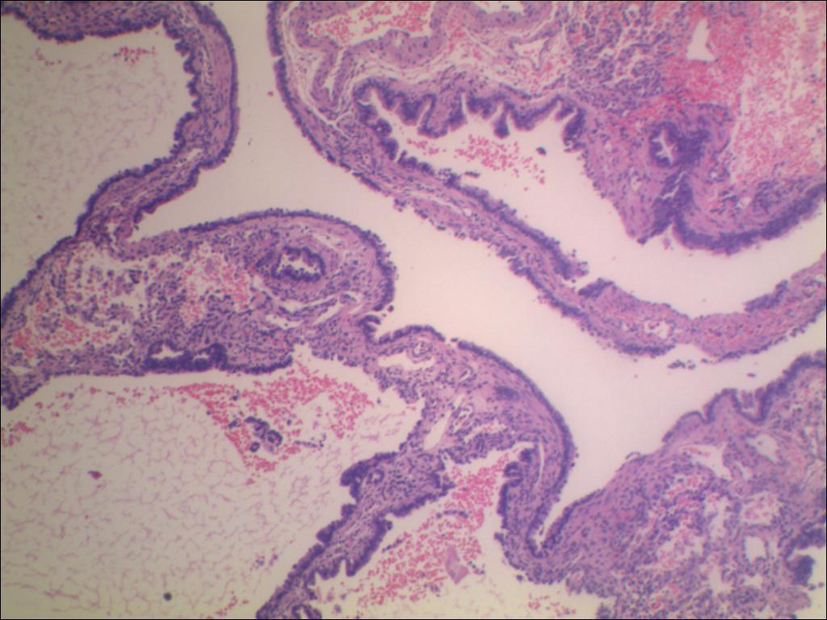

Supplement: Supplemental Digital Content [file medi-99-e20052-s003.tif]

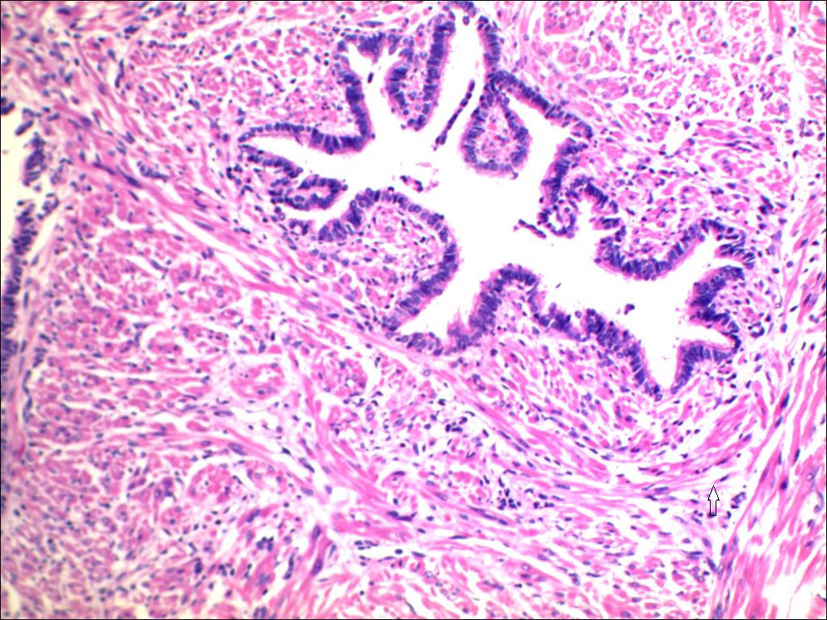

Supplement: Supplemental Digital Content [file medi-99-e20052-s004.tif]

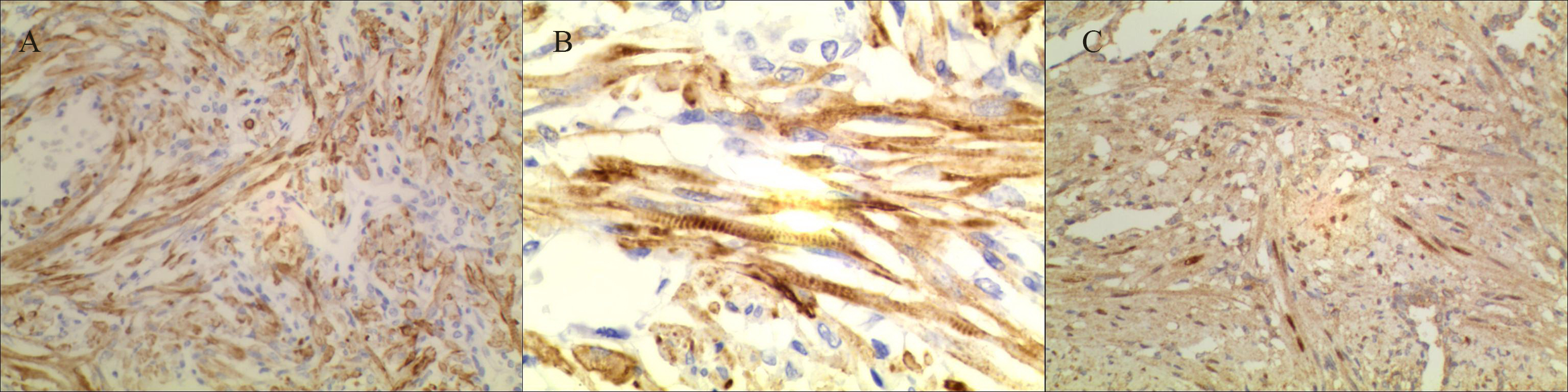

Supplement: Supplemental Digital Content [file medi-99-e20052-s005.tif]
